# miR-29a sensitizes the response of glioma cells to temozolomide by modulating the P53/MDM2 feedback loop

**DOI:** 10.1186/s11658-021-00266-9

**Published:** 2021-05-27

**Authors:** Qiudan Chen, Weifeng Wang, Shuying Chen, Xiaotong Chen, Yong Lin

**Affiliations:** 1 The Department of Central Laboratory, Clinical Laboratory, Jing’an District Center Hospital of Shanghai, Fudan University, Shanghai, 200040 China; 2 Department of Central Laboratory, Clinical Medicine Scientific and Technical Innovation Park, Shanghai Tenth People’s Hospital, Tongji University School of Medicine, Shanghai, 200435 China; 3 Department of Laboratory Medicine, Huashan Hospital, Fudan University, Shanghai, 20040 China

**Keywords:** Glioma, miRNA, miR-29a, p53, MDM2

## Abstract

Recently, pivotal functions of miRNAs in regulating common tumorigenic processes and manipulating signaling pathways in brain tumors have been recognized; notably, miR‐29a is closely associated with p53 signaling, contributing to the development of glioma. However, the molecular mechanism of the interaction between miR-29a and p53 signaling is still to be revealed. Herein, a total of 30 glioma tissues and 10 non-cancerous tissues were used to investigate the expression of miR‐29a. CCK-8 assay and Transwell assay were applied to identify the effects of miR-29a altered expression on the malignant biological behaviors of glioma cells in vitro, including proliferation, apoptosis, migration and invasion. A dual-luciferase reporter assay was used to further validate the regulatory effect of p53 or miR-29a on miR-29a or MDM2, respectively, at the transcriptional level. The results showed that miR-29a expression negatively correlated with tumor grade of human gliomas; at the same time it inhibited cell proliferation, migration, and invasion and promoted apoptosis of glioma cells in vitro. Mechanistically, miR-29a expression was induced by p53, leading to aberrant expression of MDM2 targeted by miR-29a, and finally imbalanced the activity of the p53-miR-29a-MDM2 feedback loop. Moreover, miR-29a regulating p53/MDM2 signaling sensitized the response of glioma cells to temozolomide treatment. Altogether, the study demonstrated a potential molecular mechanism in the tumorigenesis of glioma, while offering a possible target for treating human glioma in the future.

**Supplementary Information:**

The online version contains supplementary material available at 10.1186/s11658-021-00266-9.

## Introduction

Among primary brain tumors in adults, gliomas are most common, representing about 80% of malignant brain tumors [1, 2]. Of note, malignant glioma is highly aggressive, leading to the difficulty of radical resection [3]; meanwhile the 5-year survival rate of malignant glioma patients is less than 5% [4]. At present, it is well known that the malignant growth of gliomas often results from uncontrollable proliferation and dysregulated apoptosis [5]. Therefore, a deeper understanding of the malignant growth mechanism in the pathogenesis of gliomas as well as potential therapeutic targets has become an urgent issue to be solved, to finally improve the efficacy of chemotherapy and patient prognosis.

In recent years, microRNAs (miRs) have been recognized as important participants in cancer development and progression; meanwhile they have been found to be dysregulated in almost all types of cancers [6]. As a novel class of small non-coding RNAs, miRs can bind to the 3ʹ-untranslated region (3ʹ-UTR) of the mRNA and inhibit gene expression at the post-transcriptional level [7]. Recent studies have demonstrated pivotal functions of miRs in regulating common tumorigenic processes and manipulating signaling pathways in brain tumors, including gliomas [8]. Meanwhile, increasing evidence has shown that the dysregulated expression of miRs is highly relevant to glioma tumorigenesis and prognosis of patients [9]. For instance, several studies showed that, among these miRs, aberrated expression of miR-29 is strongly associated with more aggressive behavior and poorer prognosis in glioma patients [10–12]; moreover, the miR-29 family could be recognized as biomarkers for high-grade glioma [13]. Regarding the molecular mechanism, it was reported that miR-29 inhibited the malignant behaviors of glioblastoma cells via targeting DNA methyltransferases 3A and 3B (DNMT3A and 3B) [12]; also it was found to be involved in regulation of the SCAP [SREBP cleavage-activating protein]/SREBP-1[sterol regulatory element binding protein 1]) feedback loop and to modulate epidermal growth factor receptor (EGFR) signaling-driven glioma growth [14].

To date, the miR-29 family has been extensively reported to be related to a variety of activities including tumorigenesis, epigenetic modification, embryonic development and others [15]. One member of the miR-29 family, miR‐29a, was demonstrated in a recent study to activate growth and promote invasion in glioma by regulating protein kinase B (AKT) signaling and repressing translation of the transcription factor-Sox4 [16]. Moreover, another recent study showed that miR‐29a impeded stemness and tumor growth of glioma cells by regulating the platelet derived growth factor (PDGF) pathway [17] or a negative feedback loop of TNF receptor associated factor (TRAF4)/Akt signaling [18]. Notably, a previous study showed that miR-29a upregulated p53 levels and induced apoptosis dependent on p53 function, in a screen for miRs that can modulate p53 activity [19]. As one of the most widely known tumor suppressor proteins to date, p53 is always implicated in almost every cancer, including glioma [20]. p53 can not only effectively inhibit cellular growth and respond to a variety of stresses in cells [21], but also its expression is associated with pathological grade of glioma and might serve as a predictor for the prognosis of glioma patients [22, 23]. Nevertheless, the role of p53 on miR‐29a expression in glioma is still ill-defined and the internal molecular mechanism of the interaction between p53 signaling and miR-29a needs to be clarified.

In this study, we observed that miR-29a expression negatively correlated with grades of human gliomas and verified that upregulated miR-29a inhibited proliferation, suppressed migration and invasion but promoted apoptosis of glioma cells; mechanistically, miR-29a expression was induced by p53, led to aberrated expression of mouse double minute 2/4 (MDM2/4) targeted by miR-29a, and finally imbalanced the activity of the p53-miR-29a-MDM2/4 feedback loop; in the view of clinical application, miR-29a regulating p53/MDM2 signaling sensitized the response of glioma cells to temozolomide. All of the results suggested a novel molecular mechanism in glioma tumorigenesis and provided a potential target for treating human glioma in the future.

## Materials and methods

### Samples

A total of 30 human glioma tissues and 10 nontumoral tissues were gained from patients who had experienced surgical procedures at the Huashan Hospital (Shanghai, China) between January 2016 and December 2016. All samples including glioma tissues and nontumoral tissues were confirmed by histopathological examination; available resected tissues were included while tissues with preoperative radiation or chemotherapy were excluded. In addition, 6 tumor tissues from patients who were receiving temozolomide (TMZ) treatment and achieving a better response and 6 tumor tissues from patients who were receiving TMZ treatment but with no response were collected for evaluation of miR-29a, p53 and MDM2 expression. The characteristics of patients are shown in Table 1. All aspects of this study (No. 2017-087) were approved by the Ethics Committee of Huashan Hospital on January 12, 2017, in accordance with the 1964 Helsinki Declaration and its later amendments or comparable ethical standards. All patients provided written consent. A preset proportion of collected specimens were frozen in liquid nitrogen and stored at -80℃; the remaining specimens were made as formalin-fixed paraffin-embedded (FFPE) samples for hematoxylin–eosin (HE) or immunohistochemical (IHC) assessment.

**Table 1:** The association between miR-29a expression and clinicopathological characteristics of included patients

Clinicopathological characteristics	No. of patients	miR-29a expression		P
		High	Low	
WHO grade				< 0.05
I	7	6	1	
II	7	5	2	
III	7	3	4	
IV	9	3	6	
Age				0.518
< 50	14	8	6	
≥ 50	16	9	7	
Gender				0.728
Male	16	8	8	
Female	14	8	6	
Location				
Supratentorial	18	10	8	0.469
Infratentorial	12	5	7	
Tumor size				
< 3 cm	13	7	5	0.065
≥ 3 cm	17	11	6	

The median expression level of lncRNA- was used as the cutoff

### Cell culture

Human glioma cell lines U87 and U251 were respectively obtained from the American Type Culture Collection (ATCC, Manassas, USA) and National Collection of Authenticated Cell Cultures (NCACC, Shanghai, China). These cells were cultured and maintained in complete DMEM medium supplemented with 10% fetal bovine serum (FBS, Gibco, USA), in a humidified incubator of 95% air and 5% CO_2_ at 37℃ for subsequent experiments.

### Cell transfection

miR-29a mimic, miR-29a inhibitor and corresponding controls were obtained from GenePharma (Shanghai, China). Transfection was applied using Lipofectamine 3000 reagent according to the manufacturer’s protocols. For transient transfection, 50 nM RNA oligonucleotides and the determined amount of Lipofectamine 3000 were diluted in 250 µL of Opti-MEM medium. After being gently mixed and incubated for 20 min at room temperature, a complex formed and then was incubated with 1 × 10^5^ cells for 6 h. Following that, the medium was replaced with fresh medium and the cells were harvested at indicated time points after transfection. Lentivirus-induced transfection was used to generate stable expression. During cell transfection in vitro, the vectors encoding EGFP were used as an alternative to evaluate the transfection efficiency.

### Proliferation assay

In brief, stably transfected U251 or U87 cells were plated in a 96-well plate with a concentration of 2 × 10^4^/L; after cells were cultured overnight, 10 µL of CCK-8 solution (Dojindo, Japan) was added to each well on the 1st, 2nd, 3rd and 4th days, and the cells were incubated for 2 h. Then, cell proliferation was measured by detecting the absorbency at 450 nm following the manufacturer’s instructions.

### Apoptosis assay

In the study, the Annexin V-PE Apoptosis Detection Kit from Beyotime Biotechnology (Hang zhou, China) was used to evaluate cell apoptosis. The measurement was performed according to the manufacturer’s protocol. In brief, 1 × 10^5^ transfected cells were detached with 2.5% trypsin–EDTA; after centrifugation at 800 rpm for 5 min, cells were resuspended in 195 μL of Annexin V-PE 1 × binding solution. Then, the cell suspension was supplemented with 5 μL of Annexin V-PE solution, and incubated in the dark at room temperature for 15–20 min. The apoptosis rates were finally calculated based on a flow cytometer analysis (Beckman Coulter Inc., USA).

### Migration assay

Cell migration was tested in a Transwell assay following the manufacturer’s guidelines. In brief, a 200 μL cell suspension containing 2 × 10^4^ U87 or U251 cells was placed on the upper layer of a Boyden Chamber with 8-μm polycarbonate membranes and 600 μL of complete medium was added below the membrane. Then cells were incubated at 37℃ for 36 h. Cells that migrated through the membrane were fixed with 90% ethanol for 15 min followed by staining with 0.1% crystal violet solution for 5 min at room temperature. Finally, the stained cells were photographed under the microscope (Olympus, Japan) and calculated by counting from six random fields.

### Invasion assay

Cell invasion assay was performed using the 24-well Transwell chambers as previously described [24]. In brief, the chambers were precoated with Matrigel matrix (BD Biosciences, USA). A 200 μL cell suspension containing 2 × 10^4^ U87 or U251 cells was seeded on the upper chamber, and 600 μL of complete medium was added to the lower chamber. After 36 h, cells on the upper chamber were removed while cells that passed through the membrane were fixed with 4% paraformaldehyde, and then stained with 0.1% crystal violet solution. To perform the quantitative analysis, cells were photographed under a microscope (Olympus, Japan) by counting from six random fields.

### Dual-luciferase reporter assay

To predict target genes for miR-29a, the TargetScan online database (http://www.targetscan.org) was used. The DNA fragment corresponding to the 3ʹ-UTR of MDM2 mRNA was amplified by RT-PCR and cloned into the pEZX-MT01 vector (GeneCopoeia Inc., USA); in contrast, the 3ʹ-UTR of the MDM2 mutant with a binding site for miR-29a was constructed using a site-directed mutagenesis PCR method. The E-box element at − 481 bp was mutated from AUCAGC to GGUUAA using overlapping primer. 48 h after miR-29a mimic transfection, the transcription activity of the construct promoter was detected by performing a dual-luciferase reporter assay according to the manufacturer’s instructions (Promega Corporation); in the assay, the pEZX vector was used as an internal control for normalization.

### Quantitative RT-PCR (RT-qPCR)

Total RNA was extracted from the cultured glioma cells with/without transfection and tumor or nontumoral tissues using TRIzol Reagent (Invitrogen, MA, USA) according to the manufacturer’s protocols. RNA was reverse transcribed using the PrimeScript RT reagent Kit and the expression of miR-29a was tested by applying the Mir-X miRNA qRT-PCR SYBR Kit (Takara Biotechnology, Dalian, China). The expression level of MDM2 was determined using the Transcriptor First Strand cDNA Synthesis Kit and FastStart Universal SYBR Green Master (Rox, Roche, USA). The primers were designed as follows: miR-29a: 5ʹ-TACTGAAC TGTCAC GGC AGA-3ʹ(fw), 5ʹ-TGTAGTTAGCGACCTCTGCT-3ʹ(rev); MDM2: 5ʹ-ACCCTGGTT AGACCA AAGCC-3ʹ(fw), 5ʹ-TGGCACGCCAAACAAATCTC-3ʹ(rev); β-actin: 5ʹ-AGAAGGCTGGGG CTCATTTG-3ʹ(fw), 5ʹ-AGGGGCCATCCAC AGTCTTC-3ʹ(rev). All of the reactions were performed in triplicate; data were analyzed based on the classic 2^−ΔΔCt^ method.

### Western blotting

Proteins were extracted using RIPA lysis buffer supplemented with protease inhibitors and phosphatase inhibitors. A total of 20 μg proteins from each sample were separated by 10% SDS-PAGE electrophoresis; then proteins were transferred onto polyvinylidene difluoride (PVDF) membranes. The membranes were blocked with 5% BSA for 1 h at room temperature; after that, membranes were incubated with anti-p53, anti-MDM2 or anti-β-actin (1:1000; Cell Signaling Technology, USA) at 4℃ overnight. After washing three times, horseradish peroxidase-conjugated secondary antibodies were incubated for 1 h. The proteins were visualized using the SuperSignal West Pico Chemiluminescent Substrate kit (Thermo Fisher Scientific Inc., USA). β-actin was used as a loading control. All experiments were independently performed in triplicate.

### Animal procedures

Four-week-old male BALB/c nude mice were purchased from Shanghai Laboratory Animal Center (Chinese Academy of Sciences, Shanghai, China) and maintained in special pathogen free (SPF) conditions for 1 week to adapt to the environment. 5 × 10^6^ U87/miR-C (control), U87/miR-29a, or U87/miR-29a-I (inhibitor) cells were resuspended in 200 μL of PBS solution and then subcutaneously injected into two sides of the posterior flanks of nude mice. When tumors were apparently seen, mice were intraperitoneally injected with TMZ (50 mg/kg every two days). During the experiment, tumor size was continuously detected and tumor volume was accordingly calculated. After mice were sacrificed and tumors were dissected at the 4th week, hematoxylin and eosin-stained sections were used to analyze tumor formation and histological phenotype. Animal procedures were performed and granted (No. 20171074A016) by the Ethics Committee of Huashan Hospital on January 8, 2017, in accordance with institutional guidelines for the care and use of laboratory animals.

### Immunohistochemical (IHC) staining

The FFPE sections were used for IHC examination. In brief, after deparaffinizing, permeabilization of sections was carried out in citrate buffer solution followed by microwaving for 10 min; after washing twice with 1 × phosphate buffer solution (PBS), the sections were taken and incubated in 3% H_2_O_2_ for 15 min to block endogenous peroxidase activities. After washing with 1 × PBS again, sections were incubated with goat serum for 30 min, then anti-p53 (1:200) or MDM2 (1:100) primary antibody was added and incubated at 4 °C, overnight. After washing, the sections were incubated with secondary biotinylated antibody and then visualized using a streptavidin-peroxidase conjugate and diaminobenzidine kit (Zhongshan Biotech, Beijing, China). Representative images were photographed using a digital-sight imaging system (Nikon Corp., Japan).

### Statistical analysis

All statistical analysis was conducted with SPSS 19.0 software (SPSS Inc., Chicago, IL, USA). For comparison of quantitative variables among groups, one-way analysis of variance (ANOVA) or the Mann–Whitney test was used. For difference in proportions among groups, the chi-squared test was performed. Data are shown as mean ± standard deviation (SD). A two-side p-value of less than 0.05 was considered to indicate statistical significance.

## Results

### Downregulated miR-29a correlates with tumor grade in human gliomas

**Fig. 1:**
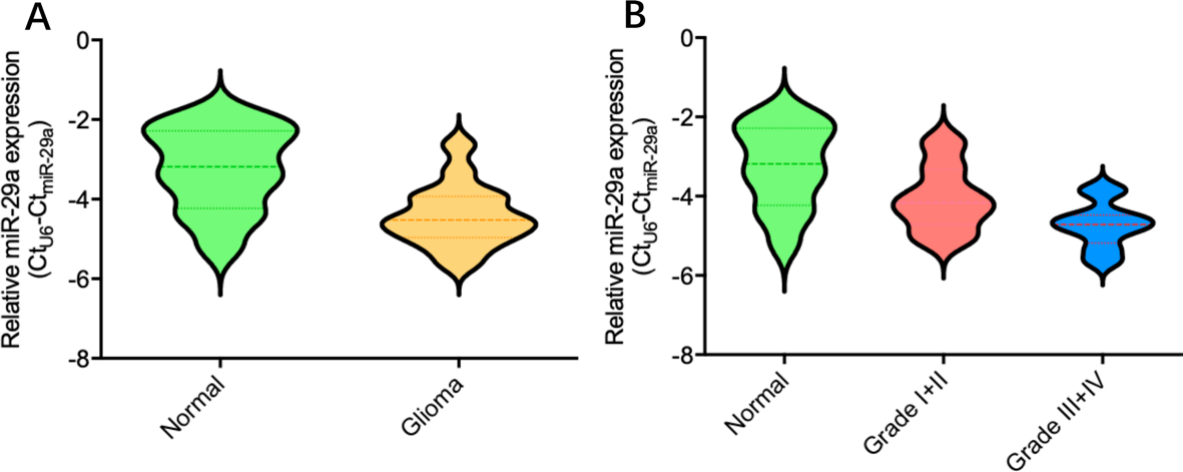
Features of miR-29a expression in glioma samples and nontumoral brain tissues. **A** miR-29a expression was decreased in glioma samples as compared to nontumoral brain samples (P = 0.0047). **B** miR-29a expression was decreased in glioma samples with high grade (III + IV) as compared to low grade (I + II) (P = 0.0212)

To investigate the features of miR-29a expression in human glioma, we collected a total of 30 resected human glioma tissues and 10 nontumoral brain tissues. Using qRT-PCR, we observed that miR-29a expression decreased significantly in the glioma samples, as compared to the nontumoral samples (P < 0.05) (**Fig. ****1****A**), consistent with reports in other cancers [25]. Moreover, miR-29a expression progressively decreased as the glioma grade increased (P < 0.05) (Fig. **1****B** and Table **1**). These results suggested the importance of miR-29a as a suppressor in tumorigenesis of neuroglial cells.

### miR-29a affects proliferation, migration, invasion and apoptosis of glioma cells in vitro

**Fig. 2:**
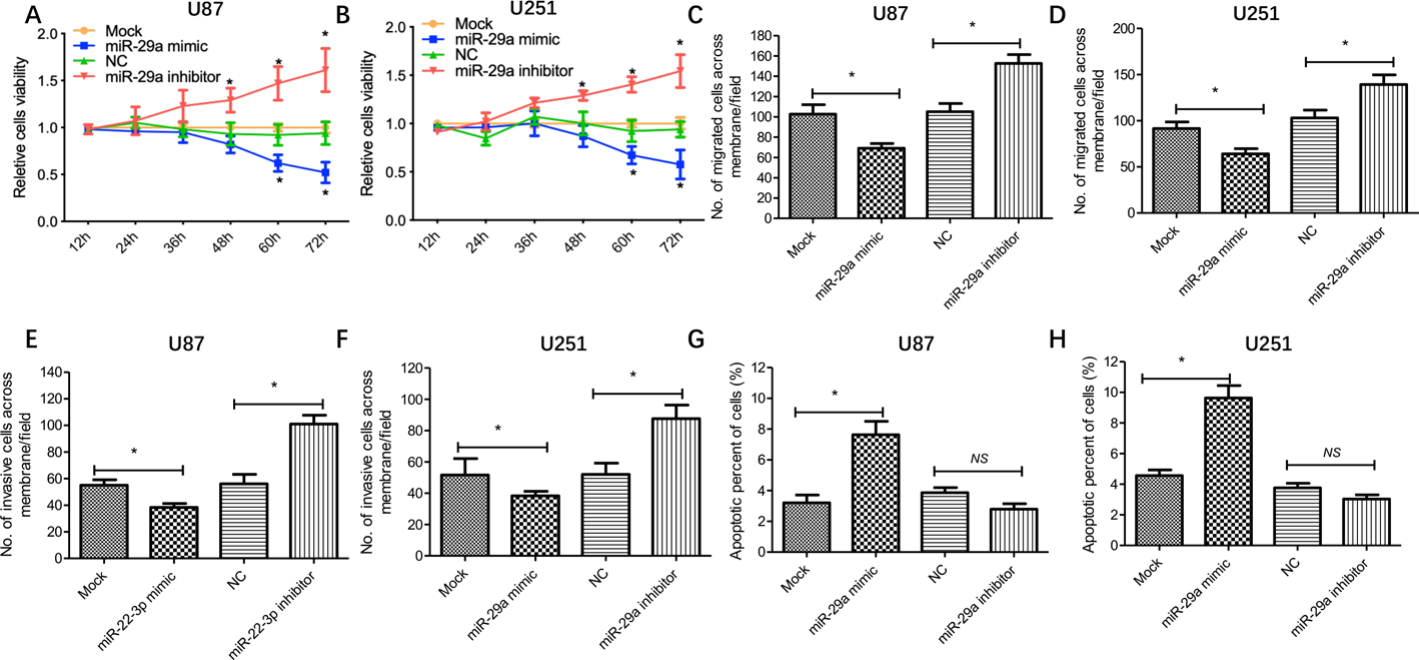
miR-29a affects glioma cell proliferation, migration, invasion and apoptosis in vitro. After miR-29a mimic, inhibitor or corresponding control treatment, proliferation (U87:A; U251:B), migration (U87:C; U251:D), invasion (U87:E; U251:F) and apoptosis (U87:G; U251:H) of glioma cells were measured by CCK-8 assay, transwell assay and flow cytometry. Results represent the mean value ± SD (n = 3). *P < 0.05

To verify the roles of miR-29a in malignant behaviors of glioma cells, including proliferation, migration, invasion and apoptosis, we evaluated the cellular functions mentioned above after miR-29a expression changed in glioma cells. As shown in Fig. 2, a significant decrease of cell proliferation was observed in glioma cells with miR-29a mimic transfection, while cell proliferation presented an increase when applying miR-29a inhibitor (Fig. 2A, B). Moreover, transwell assays showed that migration and invasion of glioma cells with miR-29a mimic transfection were significantly suppressed, while miR-29a inhibitor treatment promoted migration and invasion of these cells (Fig. 2C–F). In addition, miR-29a mimic transfection led to increased apoptosis of glioma cells, whereas miR-29a inhibitor treatment did not result in a significant change of apoptosis but showed a downregulated trend (Fig. 2G, H). Collectively, miR-29a could function as a tumor suppressor in glioma.

### miR-29a expression is induced by P53

**Fig. 3:**
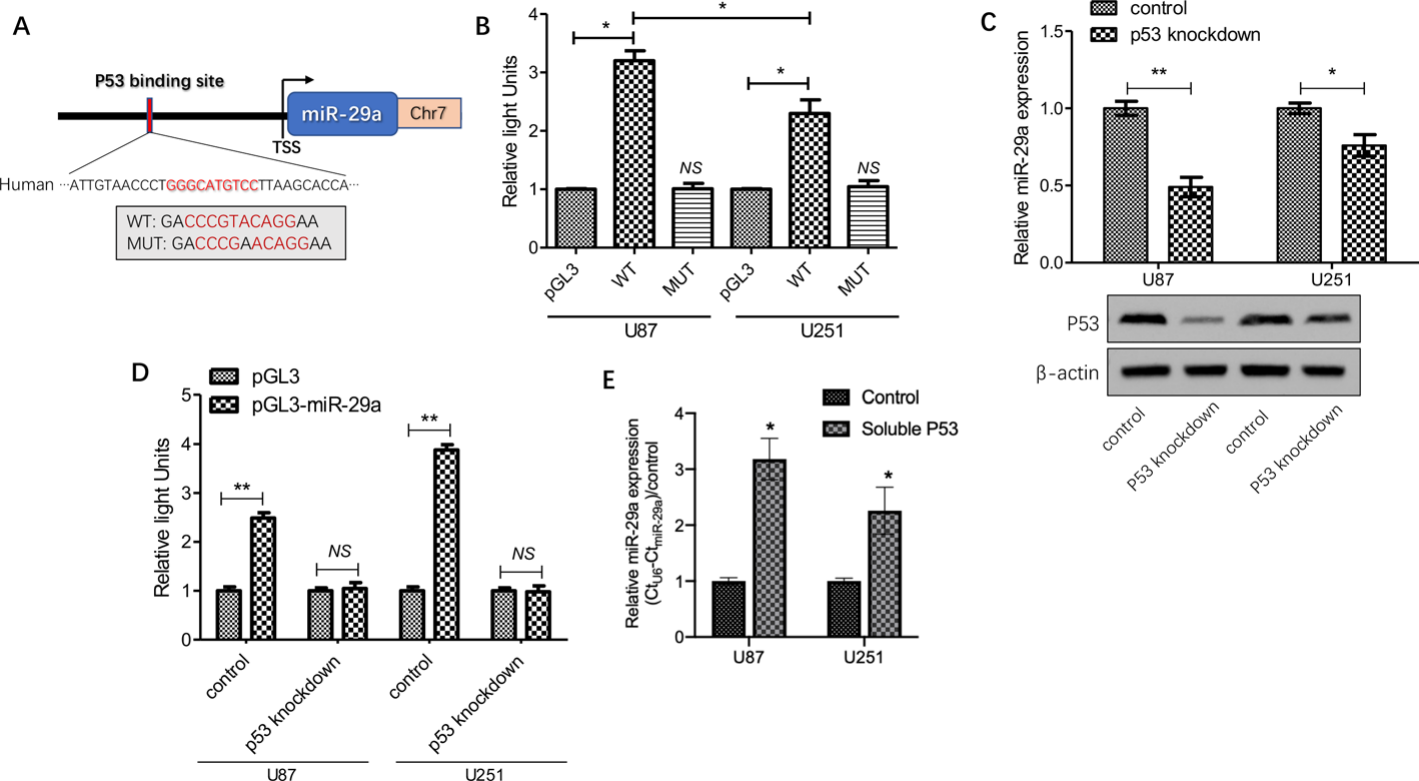
miR-29a expression is induced by P53. **A** A schematic representation showed a potential P53 binding site 2.2 kb upstream of the miR-29a TSS. **B** A sequence of the miR-29a promoter with the P53 binding site or site directed mutagenesis of the identified sequence was cloned into a pEZX-MT01 luciferase reporter vector, after transfection into glioma cells (U87 and U251). The direct regulation of P53 on miR-29a was assessed by luciferase reporter assay; the pEZX basic vector was used as a control. **C** After p53 was effectively knocked down in glioma cells (U87 and U251), miR-29a expression was detected by relative quantity RT-PCR. **D** The wild-type pEZX-miR-29a construct-transfected cells with or without p53 knockdown was treated and then the luciferase reporter activity in these cells was detected. **E** Commercial soluble p53 protein was added to culture media and miR-29a expression in glioma cells was measured. Results show the mean ± SD (n = 3). *P < 0.05; **P < 0.01

To determine whether miR-29a expression can be induced by P53, we firstly defined the miR-29a promoter using the UCSC Genome Browser (https://genome.ucsc.edu/); subsequently, we performed a screening of up to 2.2 kb upstream of the transcriptional start site (TSS) of miR-29a to find the potential P53 binding sites and identified a P53 binding site at − 806/− 820 bp upstream of the miR-29a TSS (Fig. 3A). To further identify the direct transcriptional activation of p53 on miR-29a expression in glioma cells, we cloned a segment of the miR-29a promoter containing the predicted P53 binding site or mutated site into the pEZX vector. By using dual-luciferase reporter assay, we observed that the luciferase reporter activity significantly increased in glioma cells (U87: 3.2-fold; U251: 2.3-fold, respectively) with the wild-type pEZX-miR-29a construct as compared to cells with pEZX vector transfection, while mutant pEZX-miR-29a construct transfected cells did not show a significant increase in activity. Interestingly, there was a significant difference in the increase of activity between U87 cells (wild-type p53) with the wild-type pEZX- miR-29a construct and U251 cells (mutated p53) with the wild-type pEZX-miR-29a construct (P < 0.05), suggesting that the genetic status of p53 could be an important factor for inducing miR-29a expression in glioma cells (Fig. 3B). To further confirm the role of P53 in inducing miR-29a expression, we knocked down P53 expression with lentivirus-induced transfection in glioma cells and found that miR-29a expression was significantly suppressed, as expected (Fig. 3C). In addition, we treated the wild-type pEZX-miR-29a construct-transfected cells with or without p53 knockdown and then detected the luciferase reporter activity in these cells. It was found that an increase in activity was not detected after p53 knockdown (Fig. 3D). Furthermore, we added commercial soluble p53 protein to culture media and observed also that miR-29a expression was upregulated in glioma cells (Fig. 3E). Altogether, these results strongly suggested that miR-29a expression is induced by presence of P53 in glioma.

### miR-29a directly targets MDM2 in glioma cells

**Fig. 4:**
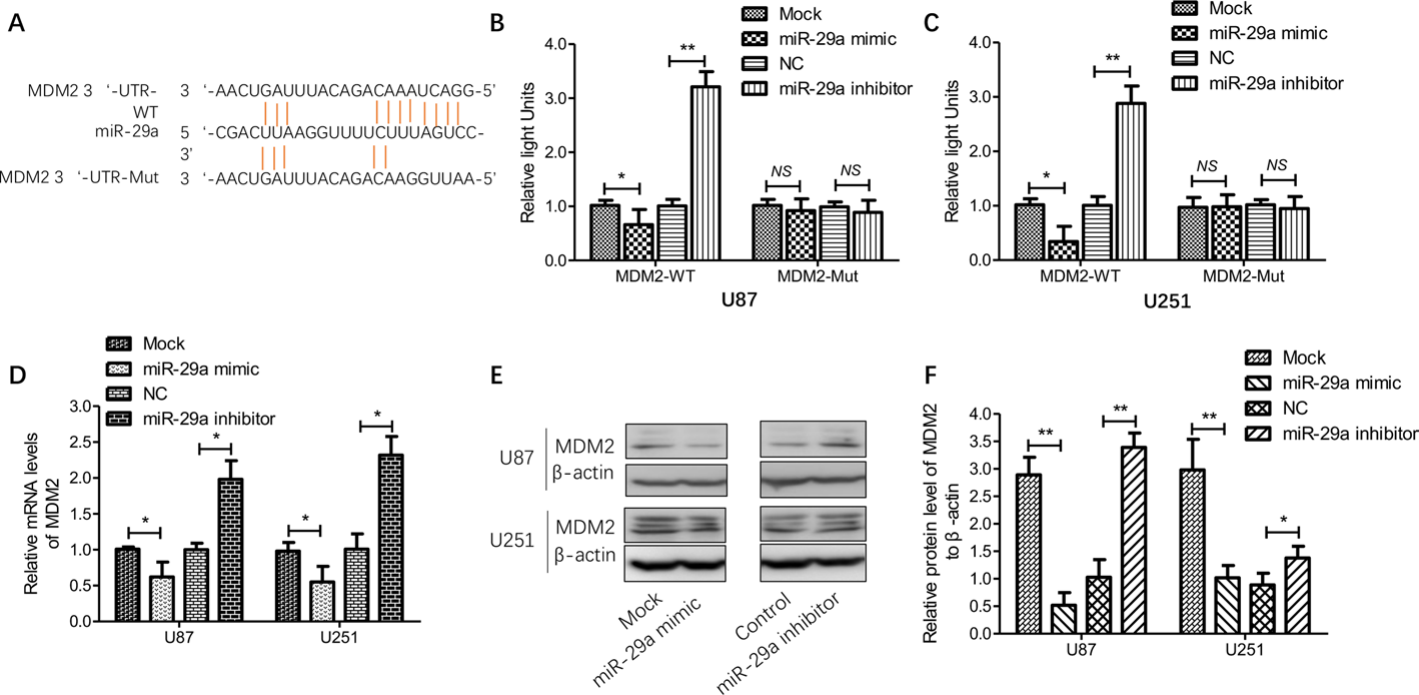
Effect of direct transcriptional regulation of miR-29a on MDM2 expression. **A** A typical representation of the putative binding site of miR-29a in the 3ʹ-UTR of MDM2. **B**, **C** Firstly, the wild-type or mutated full-length 3ʹ-UTR of the MDM2 gene was cloned into dual-luciferase reporter vectors; secondly, the vectors were co-transfected with miR-29a mimics, inhibitors or corresponding controls; finally, the activity was evaluated by luciferase reporter assay. **D** In glioma cells with miR-29a mimic, inhibitor or corresponding control transfection, relative mRNA levels of MDM2 were measured by relative quantity RT-PCR. **E**–**F** In glioma cells mentioned above, protein levels of MDM2 were detected by western blotting. Results are expressed as the mean ± SD (n = 3). *P < 0.05; **P < 0.01

MDM2 interaction with P53 leads to the latter’s degradation. Many studies have reported molecular alterations of the p53-MDM2 pathways in human glioma; hence disrupting the MDM2-p53 interaction is a promising strategy to treat glioma [26–28]. It was recently reported that reciprocally transcriptional regulation via a negative feedback loop exists between p53 and MDM2 [27]. To further verify the relationship between miR-29a and p53-MDM2 pathways in glioma, we firstly defined whether miR-29a directly targeted MDM2, the other important element of p53-MDM2 pathways, by screening the targets of miR-29a based on the TargetScan database and identified MDM2 as a potential target of miR-29a (Fig. 4A). Next, we cloned predicted wild-type (WT) or mutated (Mut) full-length 3ʹ-UTR of the MDM2 gene into a dual-luciferase reporter plasmid and then co-transfected it with miR-29a mimics, inhibitors or controls into glioma cells. It was found that the luciferase activity decreased after co-transfection of miR-29a mimics in glioma cells (U87 and U251), whereas the activity relatively increased in cells co-transfected with miR-29a inhibitors (Fig. 4B–C). Then the transcriptional and protein levels of MDM2 in cells with miR-29a mimics, inhibitors or control transfection were detected, and the results showed that miR-29a mimics significantly reduced the mRNA and protein levels of MDM2 while miR-29a inhibitors significantly increased the levels of MDM2, as expected (Fig. 4D–F). These findings further verified that miR-29a negatively regulated MDM2 expression via directly targeting MDM2 in glioma cells.

### P53-miR-29a-MDM2 positive feedback loop sensitized the response of glioma cells to temozolomide

**Fig. 5:**
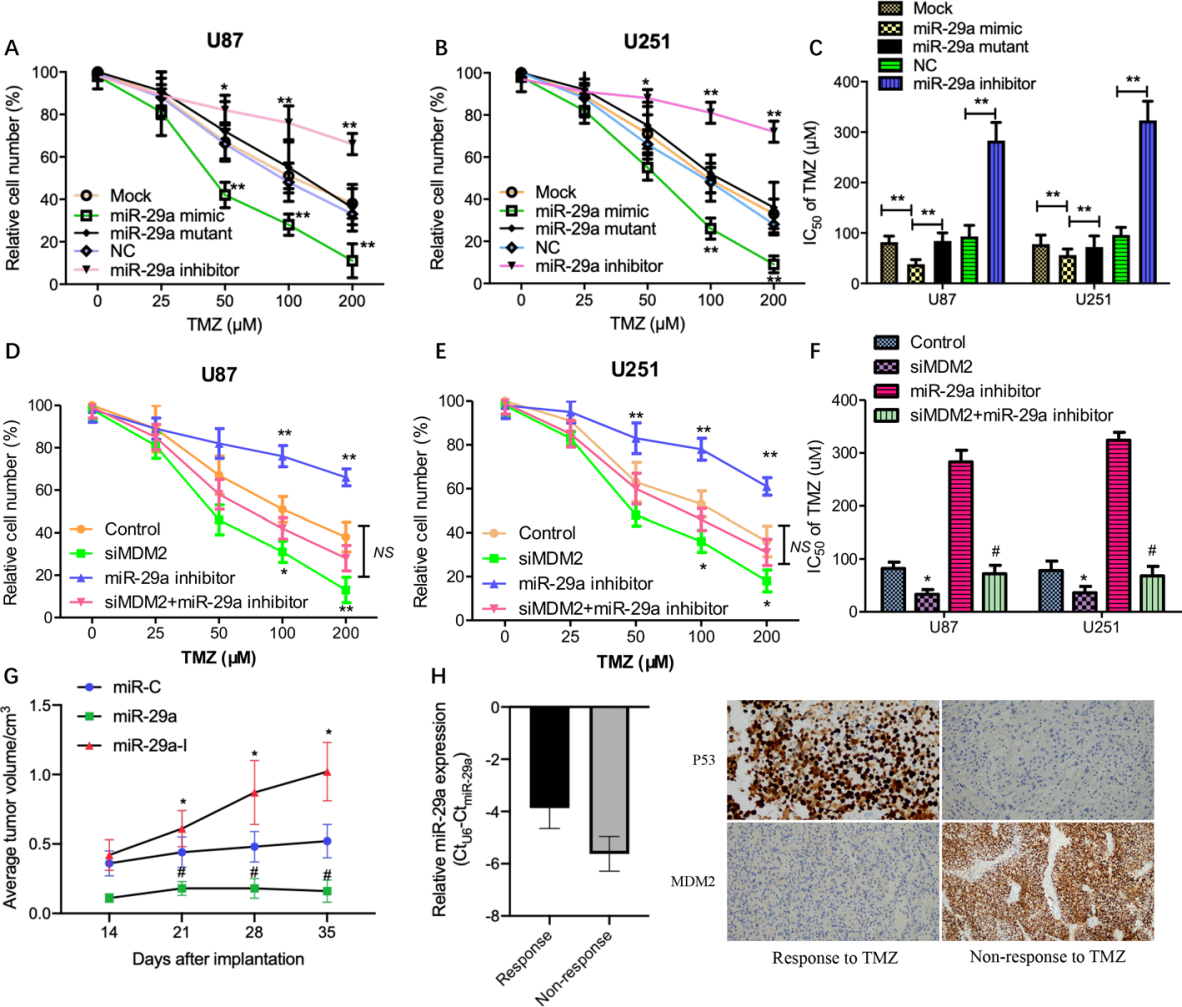
P53-miR-29a-MDM2 positive feedback loop sensitized the response of glioma cells to temozolomide. **A**, **B** After using a serial concentration of TMZ to treat glioma cells with miR-29a mimic, inhibitor or corresponding control transfection, cell viability was detected by CCK-8 assay. **C** IC_50_ for TMZ was determined in glioma cells mentioned above. Results show the mean ± SD (n = 3). *P < 0.05; **P < 0.01. **D**, **E** After MDM2 knockdown followed by miR-29a inhibitor treatment, cell viability was detected by CCK-8 assay; meanwhile IC_50_ for TMZ was determined in these cells (**F**). *P < 0.05 vs control; ^#^P < 0.05 vs miR-29a inhibitor. U87 cells stably expressing miR-C (control), miR-29a, or miR-29a-I (inhibitor) were resuspended in 200 μL of FBS-free DMEM medium and then subcutaneously injected into each side of the posterior flanks of nude mice (n = 6). The tumor growth curve in vivo was analyzed (**G**). Furthermore, tumor tissues from patients receiving TMZ treatment and achieving a better response and from patients receiving TMZ treatment but without a response were collected for the evaluation of miR-29a, p53 and MDM2 expression by RT-PCR and IHC (**H**)

The results described above suggested that a P53-miR-29a-MDM2 positive feedback loop exists in neuroglial cells; however, the downregulation of miR-29a expression may retard the feedback loop, leading to the dysfunction of P53 as a tumor suppressor in glioma cells. Recently, it was demonstrated that activation of MDM2/p53 signaling facilitates the resistance of glioma cells to temozolomide (TMZ) [29]. Hence, we addressed the question whether disruption of the identified P53-miR-29a-MDM2 feedback loop can sensitize the response of glioma cells to TMZ. Firstly, we observed the sensitivity change of glioma cells to TMZ following transfection with miR-29a mimics, inhibitors or corresponding controls. It was found that miR-29a mimic accelerated the inhibition rate of viable cells in glioma cells and enhanced the sensitivity of glioma cells to TMZ, while the miR-29a mutant did not display such effects; in contrast, miR-29a inhibitor treatment showed the opposite change (Fig. 5A–B); meanwhile the results for the half maximal inhibitory concentration (IC_50_) of TMZ treatment in glioma cells showed that the relative resistance of glioma cells with miR-29a mimic treatment to TMZ decreased but there was no significant change in glioma cells with miR-29a mutant, while that of glioma cells with miR-29a inhibitor increased, as compared to the corresponding controls (Fig. 5C). Next, we investigated whether the inhibition of MDM2 can reverse miR-29a inhibitor treatment-induced TMZ resistance in glioma cells. After MDM2 knockdown followed by miR-29a inhibitor treatment, it showed that knockdown of MDM2 dramatically reversed miR-29a inhibitor treatment-mediated drug resistance to TMZ, leading to a decreased number of viable cells (Fig. 5D–E) and a reduced IC_50_ of TMZ (Fig. 5F). In addition, to further investigate whether miR-29a affects tumor growth in vivo, a subcutaneous transplantation model of human glioma U87 cells was used. The results showed that, compared to the miR-C group (control), the tumor growth of the miR-29a group was significantly increased while the growth of the miR-29a-I (miR-29a inhibitor) group was decreased in the TMZ treatment condition (Fig. 5G). Furthermore, we observed higher expression levels of miR-29a and p53 and lower expression of MDM2 in glioma tissues from patients who had received TMZ treatment and achieved a better response (response to TMZ) than from those who had received TMZ treatment but without a better response (non-response to TMZ) (Fig. 5H). Based on the above data, it was inferred that miR-29a could play an important role in resistance of glioma cells to TMZ, possibly associated with p53/MDM2 signaling.

## Discussion

Mounting miRNAs have been demonstrated to be involved in the biological process of glioma [28], and aberrant expression of miRNAs is closely associated with tumor growth, stemness and prognosis in glioma through regulating glioma cell activation, persistent neuroinflammation, and abnormal macrophage polarization [9, 31, 32]. Dysregulation of miRNAs often activates glioma cells, promotes the neuroinflammatory response, and mediates macrophage polarization in the brain [33]. It has been discovered that one endogenous microRNA (miRNA) can upregulate its own expression by binding its promoter [34]. Notably, as important cytoplasmic regulators of gene expression, miRNAs can also exert specific nuclear functions during the miRNA-guided transcriptional regulation of gene expression [35]. Recently, it has been recognized that miR-29a plays key roles in multiple biological processes in human cancers [36, 37]. For example, some studies have shown that miR-29a may play a role in glioma tumorigenesis by regulating the PDGF pathway [17] and targeting TRAF4 [38]. However, a better understanding of the molecular mechanisms of miR-29a involved in initiation and development of glioma is still urgent.

In this study, we found downregulated miR-29a expression in glioma tissues as compared to nontumoral brain tissues; at the same time it was negatively correlated with tumor grade. Moreover, we confirmed that miR-29a inhibited the malignant behaviors, including proliferation, migration and invasion, of glioma cells in vitro. The results encouraged us to explore the mechanism of aberrated expression of miR-29a in glioma cells. It has been reported that numerous non-coding RNAs can be regulated by the most important tumor suppressor, p53, through creating a complex network of pathways that influence each other [39]; moreover, tumor-suppressive functions of p53 can be induced by several miRNAs [40]. Herein, we explored the possibility of miR-29a expression regulated by p53 and, as expected, verified the direct regulation of miR-29a expression by p53 at the transcriptional level through performing a dual-luciferase reporter assay, which will achieve a better molecular understanding of the interactions between p53 and microRNAs in initiation and development of glioma.

As a tumor suppressor, p53 plays a critical role in preventing tumorigenesis [41] and contributing to resistance to therapies in glioma [42, 43]. However, p53 signaling is often modulated by a number of factors, including MDM2 and other regulators [44]. Among these regulators, MDM2 can interact with p53 and meanwhile form an auto-regulatory feedback loop as a key negative regulator of p53 protein [45]. The expression of MDM2 in human cancers is often at a high level, which promotes tumor cell proliferation [46, 47]. Interestingly, we identified MDM2 as a direct target gene of miR-29a by dual-luciferase reporter assay and miR-29a downregulated MDM2 expression in the study. P53 and MDM2 have both been reported to be dysregulated through an auto-regulatory feedback mechanism and finally promoted tumor progression in many cancers [48]. Notably, miRNAs can directly regulate the transcriptional level of p53 or MDM2, leading to tumor suppression [49]. In the present study, we observed that P53 induced miR-29a expression and upregulated miR-29a led to the downregulation of MDM2, finally enhancing P53 expression in glioma cells. Moreover, higher expression levels of miR-29a and p53 and lower expression of MDM2 were observed in clinical glioma tissues from patients with response to TMZ, compared to those without response to TMZ. All of these findings strongly suggested the possible existence of a P53-miR-29a-MDM2 feedback loop in glioma cells, which may play a pivotal role in chemotherapeutic resistance in human glioma.

There are some limitations of the current study. As is well known, p53 protein has a very complex network of activity [50]. In the study, we did not provide a mechanistic interpretation of how p53, MDM2, and miR-29a interact, especially regarding the crosstalk between the status of p53 (wild-type vs mutated) and miR-29a. Secondly, in clinical practice, intra-tumor heterogeneity of glioma may impact the prognosis of patients with chemotherapeutic treatment [51]. Findings from the study were based on traditional immortalized cells lines, which may be not faithful representations of clinically relevant human disease. The shortcoming will be rectified by using patient-derived xenograft models or more cell lines in the future. Nevertheless, our findings support an important potential role for miR-29a in glioma, which is not only a suppressor that leads to a significant decrease in cell proliferation, migration and invasion as well as a tangible increase in cell apoptosis, but also is a key participant of the recognized p53-MDM2 autoregulatory feedback loop, suggesting a correlation between the p53-miR-29a-MDM2 feedback loop and malignant behaviors of glioma cells. We have identified the existence of a p53-miR-29a-MDM2 feedback loop in glioma cells, but this does not rule out other molecules which contribute to p53 suppressor activity in other studies, shown in a schematic diagram of miR-29a involvement in various pathways in glioma based on our study and other works (Additional file 1: Fig. S1). However, considering recent advances in miRNA-mediated therapy in cancers, miR-29a seems to be a potential evaluable target that could be exploited in glioma patients with dysregulated p53-MDM2 autoregulatory activity.

## Supplementary Information

**Additional file 1: Fig. S1**. The inhibition of malignant proliferation, stemness development and etc. in glioma cells.

## Data Availability

The available datasets included in the current study can be obtained from the corresponding author on reasonable request.

## Abbreviations

miR: MicroRNA
3ʹ-UTR: 3ʹ-Untranslated region
DNMT3A/B: DNA methyltransferases 3A and 3B
SREBP-1: Sterol regulatory element binding protein 1
SCAP: SREBP cleavage-activating protein
EGFR: Epidermal growth factor receptor
PDGF: Platelet derived growth factor
TRAF: TNF receptor associated factor
MDM2/4: Mouse double minute 2/4
TMZ: Temozolomide
FFPE: Formalin-fixed paraffin-embedded
HE: Hematoxylin–eosin
IHC: Immunohistochemistry
ATCC: American type culture collection
FBS: Fetal bovine serum
CCK-8: Cell Counting Kit-8
DMEM: Dulbecco’s Modified Eagle’s Medium
RT-PCR: Reverse transcription-polymerase chain reaction
RIPA: Radioimmunoprecipitation assay
PVDF: Polyvinylidene difluoride
SPF: Special pathogen free
PBS: Phosphate buffer solution
