# Supplementary figures and images for: miR-29a sensitizes the response of glioma cells to temozolomide by modulating the P53/MDM2 feedback loop
Source: Cell Mol Biol Lett. 2021 May 27;26:21. doi: 10.1186/s11658-021-00266-9 (PMC8161631; doi:10.1186/s11658-021-00266-9)

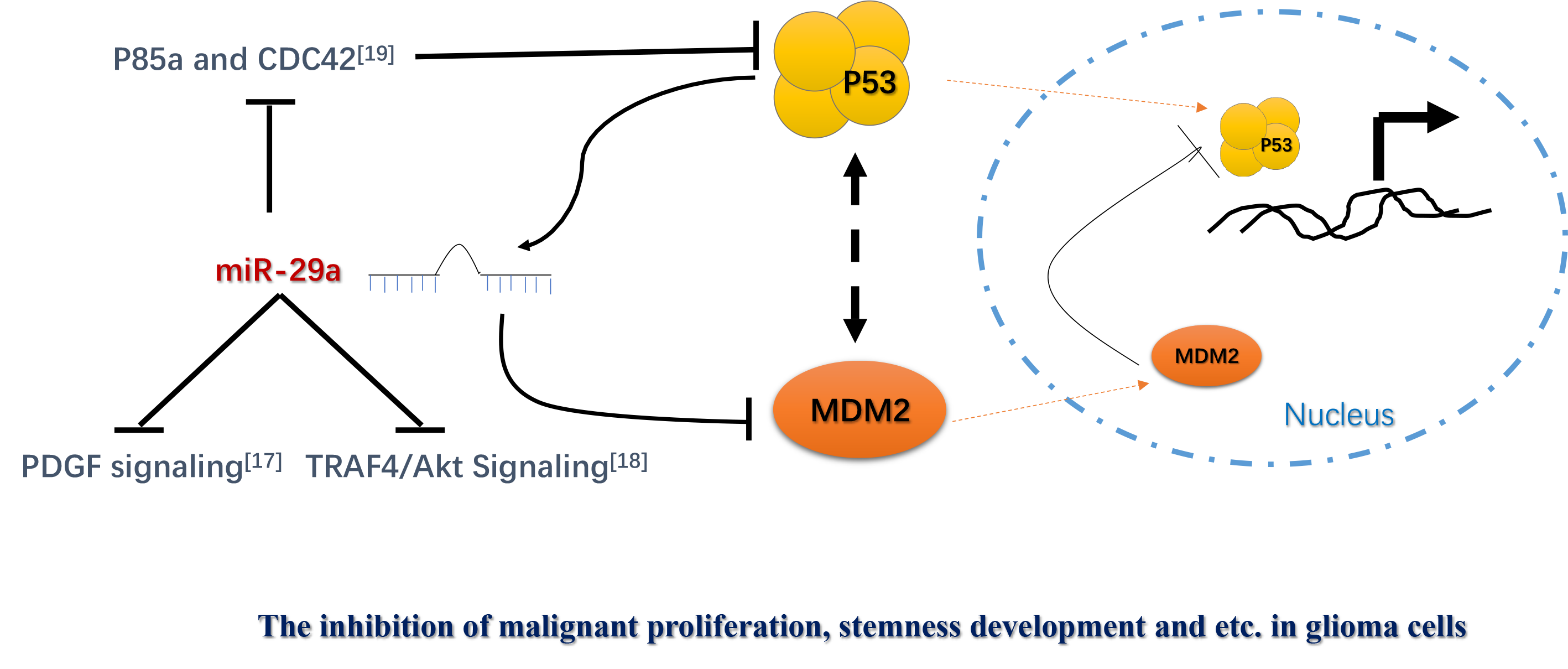

Supplement: Supplementary file 1 — Additional file 1: Fig. S1. The inhibition of malignant proliferation, stemness development and etc. in glioma cells. [file 11658_2021_266_MOESM1_ESM.tif]
